# Effectiveness of Video Modeling in Improving Technical Skills in Young Novice Basketball Players: A Quasi-Experimental Study

**DOI:** 10.3390/children10040687

**Published:** 2023-04-04

**Authors:** Amayra Tannoubi, Ibrahim Ouergui, Medina Srem-Sai, John Elvis Hagan, Frank Quansah, Fairouz Azaiez

**Affiliations:** 1 High Institute of Sport and Physical Education of Kef, University of Jendouba, El Kef 7100, Tunisia; 2 Group for the Study of Development and Social Environment, Faculty of Human and Social Science of Sfax, Sfax 3000, Tunisia; 3 Research Unit: Sports Science, Health and Movement, UR22JS01, University of Jendouba, El Kef 7100, Tunisia; 4 Department of Health, Physical Education, Recreation and Sports, University of Education, Winneba P.O. Box 25, Ghana; 5 Department of Health, Physical Education and Recreation, University of Cape Coast, Cape Coast PMB TF0494, Ghana; 6 Neurocognition and Action-Biomechanics-Research Group, Faculty of Psychology and Sports Science, Bielefeld University, Postfach 10 01 31, 33501 Bielefeld, Germany; 7 Department of Educational Foundations, University of Education, Winneba P.O. Box 25, Ghana; 8 Department of Education, Higher Institute of Sport, and Physical Education of Gafsa, University of Gafsa, Gafsa 2100, Tunisia; 9 Postgraduate School of Public Health, Department of Health Sciences, University of Genoa, 16126 Genoa, Italy

**Keywords:** training, skill acquisition, performance improvement, motor learning

## Abstract

(1) Objective: This is a quasi-experimental study that investigated the effect of four weeks of training sessions using video modeling (VM) on individual and collective technical skills in young novice basketball players. (2) Method: 20 players were equally assigned to either a control group (CG, *n* = 10; 12 ± 0.7 years) or a video modeling group (VMG, *n* = 10; 12.5 ± 0.5 years; visualizing videos before each session) were assessed before and after the four-week training period using the Basketball Skill Test of the American Alliance for Health, Physical Education, Recreation and Dance for individual techniques and three vs. three small-sided games for collective aspects. (3) Results: For the passing test, VMG induced higher performance than CG (*p* = 0.021; *d* = 0.87). For offensive balls post-intervention, higher values were recorded for VMG compared to CG (*p* = 0.003; *d* = 1.81). In addition, the number of attack balls index post-intervention was higher for VMG compared to CG (*p* = 0.001; *d* = 0.28). For losing the ball, VMG induced lower values than CG after the training intervention (*p* < 0.001; *d* = −3.23). The efficiency index was higher post-training compared to pre-training for VMG (*p* = 0.013; *d* = 1.24). (4) Conclusion: The study highlighted the importance of using video modeling as an effective strategy to improve technical skills and collective performance in novice young basketball players.

## 1. Introduction

Technology has rapidly evolved and is being incorporated into various fields of investigation including sports [1,2]. Amongst the different areas of technology, digital video modeling, which mainly focuses on social and observational learning theory [3,4] is increasingly attracting interest in varieties of sports studies [5,6,7]. As a way of frequent manipulation [8], video modeling (VM) including demonstration, video feedback, and athletes’ movement allows self-examination and self-learning to improve athletes’ motor skills [9,10,11]. Moreover, it has been revealed that most of the information that reaches the brain is acquired through the eyes/visualization [12,13]. From this perspective, various studies have suggested that VM is an effective strategy that induces learning improvements and enhances athletic movements such as gymnastics skills [9,14,15], snatch movement in weightlifting [16], tennis service [17], basketball shooting form [18,19,20], and basketball tactical skills [20,21,22,23]. This is particularly true, as digital environments allow coaches to use videos to analyze athletic movements, evaluate their team performance, and adjust their collective strategies and individual technical skills to enhance their performance [5,24]. 

VM allows players to see and analyze the correct techniques of a skill, which can be difficult to learn through only verbal instruction. Additionally, VM allows players to observe and learn from the mistakes of others [25] and this can be especially beneficial for beginners who may not be aware of common mistakes or misconceptions about a skill [26,27]. Further, by watching expert athletes’ performances on video, young beginning players can identify areas of improvement and focus their training on specific skills. Furthermore, VM can enhance collective performance through team coordination, dynamics, and communication improvements which allow players to recognize how their actions affect the performance of their teammates [28,29].

Basketball is one of the most popular team sports worldwide, requiring complex technical and tactical skills for success [30,31]. Previous studies have recommended VM as a powerful strategy that attracts both players and coaches through its effectiveness to reinforce the performance of basketball players [20,22,32] individually, and as a team [5,26,33]. This technique can be applied to basketball players at all levels, from youth and novice leagues to the professional level [20,34,35]. Because young athletes often devote more attention to video demonstrations and are easily attracted by images in motion, VM has been recommended in the literature [36]. By watching footage of themselves, players can see how they move and react on the court and adjust their technique and positioning [37] whilst watching the footage of other players, and this practice can help them to learn from the best and pick up new moves and strategies [38].

Despite the numerous benefits reported by previous studies on the use of VM in the teaching of basketball skills, using this technology has been limited to a few basketball techniques such as the free throw [39] or shooting [40], with a focus only on students’ performance [18,22,39]. However, most of these previous studies have been conducted with experienced adult athletes, leaving a gap in the literature regarding the effectiveness of video modeling for young, novice athletes. Furthermore, previous studies have primarily focused on the immediate effects of video modeling, with limited research on its long- and medium-term effects on skill improvement and transfer [41]. Therefore, the current study expands on previous studies by exploring the effectiveness of video modeling as a teaching tool for novice young basketball players and providing insight into the potential long-term benefits of this approach. Despite the growing usage of video modeling in teaching physical education practical lessons, research on how this medium of instruction can appropriately be integrated to improve technical–tactical elements during physical education is limited [42].

To date, and to the best of our knowledge, no study has investigated the effect of VM on the individual and collective performance of young basketball players. Thus, the general purpose of the present study was to examine the effects of adding video modeling during four weeks of a basketball training program for young novice players, with the specific objectives of assessing the effects on individual technical skills, assessing the impact on collective game performance measures, and comparing the performance of the video modeling group to the control group. It was hypothesized that adding VM to a habitual basketball training program would improve the individual’s technical skills and the volume of play of young novice basketball players.

## 2. Materials and Methods

### 2.1. Participants

This is a quasi-experimental study design that examined the effect of adding VM to a habitual training program for four weeks on individual and collective basketball performance. An a priori power analysis was performed using the G*Power software (Version 3.1.9.4, University of Kiel, Kiel, Germany) and the F-test family (ANOVA: repeated measures, between–within interaction). The analysis revealed that a minimum sample size of 16 participants would be adequate to detect differences (effect size f = 0.40, α = 0.05) with an actual power of 85.08%. Twenty (20) young players volunteered to participate in the present study. They were equally and randomly assigned to either control group (CG, *n* = 10; Mean ± SD: age: 12 ± 0.7 years; body mass: 47 ± 11 kg; height: 153 ± 10.5 cm) and a video modeling experimental group (VMG, *n* = 10; Mean ± SD: age: 12.5 ± 0.5 years; body mass: 51 ± 16 kg; height: 158 ± 9.5 cm) using the function of Microsoft Excel software (Table 1). The subjects were recruited from a basketball regional team, with one year of experience. The players were regularly training four sessions per week, with each session lasting 90 min. The inclusion criteria for this study were: (1) athletes should be novice basketball players from the same club and participating in the same basketball training program, (2) aged from 11 to 14 years, (3) no more than one year of basketball experience, (4) they had no injuries or medical restrictions. The exclusion criteria were: (1) players with a good previous basketball training experience or participating in school or another club team for more than one year, (2) players who are not currently participating in a training program that might interfere with the study, (3) any significant medical problems or injuries that would affect their ability to participate in a basketball training program.

### 2.2. Measures

#### 2.2.1. The Basketball Skill Test-American Alliance for Health, Physical Education, Recreation and Dance (AAHPERD)

One of the most widely adopted basketball skills test batteries was developed by the American Alliance for Health, Physical Education, Recreation and Dance (AAPHERD, American Alliance for Health, 1984). The AAPHERD test battery consists of four separate tests assessing the most common basketball skills: Passing and recovering the basketball accurately while moving, Speed Shot Shooting Test, Handling and Dribbling the ball while moving Test, and Defensive movement Skill Test [43]. In summary, these tests record successful passes while moving to different targets in 30 s on two trials (Passing Test); successful shots from different spots in 60 s × two trials (Shooting Test); elapsed time to cover a specific circuit while dribbling in two trials (left hand and right hand, Dribbling Test); and defensive movement with sliding steps without crossing the feet (Defensive Movement Test).

The scoring system for AAHPERD is as follows:

- For the passing test: two points were recorded for each chest pass that hit the center of the target, one point for a ball that touched between the targets, and 0 points for a ball that contacted below or above the targets.
- For the Speed Shot Shooting Test: two points were awarded for each basket scored, one point for each ball hitting the ring, and zero points for each air ball.
- For the dribbling test: the player established two trials in a zigzag dribble circuit placed in the restricted area. The experimenter should record the time elapsed in each trial (left hand and right hand).
- For the defensive movement test: The player was instructed to move from side to side in a circuit of eight cones in the restricted area that are placed in a zigzag form. The time elapsed during the two trials was recorded.

The intra-class correlation coefficient (ICC) for the test–retest trial for the present study was 0.91, 0.83, 0.79, and 0.79 for passing, shooting, dribbling, and defending, respectively.

#### 2.2.2. Using 3 vs. 3 Small-Sided Games

A 3 vs. 3 small-sided game in a half-court with only one hoop, excluding the lateral lanes (14 × 9 m) in order to guarantee a better interaction between players (42 m²) and the width per player near the basket (1.5 m²) was performed [44]. The game consisted of three blocks of 4 min with at least 2 min of passive rest in between [44,45]. All International Basketball Federation (FIBA) rules were respected, except for time-outs and free throws. Any personal foul committed in a team resulted in losing the ball possession, and the other team regained possession by throwing the ball from the sideline. After each basket scored, the team that conceded the points puts the ball back into play from the sideline too. Coaches verbally encouraged players in a similar way to maintain a high effort level and replaced any ball that was thrown out of play if necessary. A total of 18 bouts were video recorded and subsequently analyzed by the same investigator. Additionally, to guarantee the transparency of our results, the videos were evaluated twice by the same investigator within 7 days of interval.

A Team Sport Assessment Procedure (TSAP) has been previously reported to provide teachers and coaches with an objective tool that allows them to assess the offensive performance of students and players in different games and team sports [46]. In the present study, specific player behaviors, i.e., conquering the ball (CB), receiving the ball (RB), losing the ball (LB), offensive ball (OB), successful shot (SS), attack balls (AB), the volume of play (PB), and the efficiency index) were observed and recorded during the 3 vs. 3 small, sided game for further analysis. Additionally, the sum of different skills scores was calculated to determine different offensive indices [(i.e., number of attacking balls and volume of play (the number of times the player has gained possession)], and then the efficiency index [46] that are all calculated as follows:

- The number of attacking balls (AB): AB = OB + SS
- The volume of play (PB): CB + RB
- The efficiency index = (CB + AB)/ (10 + LB) or (CB + OB + SS)/(10 + LB)

ICC for test–retest trial for the present study was 0.98 for CB, RB, and LB, 0.92 for OB, 1 for SS, 0.96 for AB, 0.97 for PB, and 0.95 for efficiency index.

### 2.3. Procedures

Following adherence to the last Declaration of Helsinki, the protocol was fully approved by a local research ethics committee of the Higher Institute of Sport and Physical Education of Kef, University of Jendouba, Kef, Tunisia, with reference number (n° 050/2022) dated 14 December 2022. Afterwards, permission was sought from the management and coaches of the basketball regional team to allow their players to be selected to participate in the study. After obtaining a complete overview of the aims, advantages, and potential risks associated with the investigation, players and their parents signed a written informed consent/assent form.

Pre- and post-test were conducted to measure the technical performance changes across the four weeks of intervention within young novice basketball players. The assessments were conducted inside a basketball court with each assessment session lasting approximately 90 min.

This experiment was conducted in an indoor basketball court respecting the FIBA regulatory dimensions using an official size 6 ball, the dimensions of the court were 28 × 15 m, the basket was placed at a height of 3.05 m from the ground and the ring had a circumference of 0.46 m.

The pre-intervention testing session was conducted one week before the start of the training program, whereas the post-intervention assessment was conducted 48 h after the end of the program.

The assessments were administered by two trained research assistants, who were not informed of the group assignment. Athletes were assigned to VMG or a CG that performed its habitual training without any intervention. For the VMG, the intervention consisted of watching a short pre-training video sequence for four weeks (4 sessions/week) lasting approximately 3 min 30s. Ten (10) minutes before the start of each training session, the entire VMG joined a meeting room to watch video footage of a professional basketball player practicing one of the basic basketball skills according to the planned training session schedule by the team’s technical director. There are several potential benefits to choosing a professional player as a role model for new athletes. In fact, professional athletes are typically highly skilled in their sport and have a wealth of experience that can be valuable for new athletes to develop their skills in a more realistic and understandable way [47,48,49].

The experimenter did not influence the team’s practice schedule that was set in advance, and it was conducted using a Lenovo laptop computer (PC0MFE53) placed 30 cm away from the participants. The viewing angle of the screen was approximately 45° and the video was played via the VLC media player. Throughout the video session, the player could ask the coach to stop the sequence, reverse it, or ask for an explanation. After watching the video, the VMG joined the rest of the team to begin the training session. All training sessions during the intervention period were the same for both the experimental and control groups. The video sequences and exercises used in the training sessions were not similar to the exercise modality used in the tests to avoid any possible learning effects that could influence the results at the end of the intervention period. Before and after the four-week intervention phase, both VMG and CG were assessed for individual and collective basketball technical skills. Specifically, for individual technical skills (i.e., passing, shooting, dribbling, and defending) using AAHPERD [43]. For collective basketball skills, athletes were subjected to 3 vs. 3 basketball small-sided games (SSG) [45,50], video recorded, and subsequently analyzed to determine various technical–tactical aspects (i.e., conquering the ball, receiving the ball, losing the ball, offensive ball, shooting success, attacking balls, the volume of play, and the efficiency index) [46]. Before each training session, 15 min of standardized warm-up session was performed consisting of regular runs, sprints, jumps, stops and technical circuits using balls.

### 2.4. Statistical Analysis

Statistical analyses were performed using SPSS version 20.0 statistical software (IBM Corps., Armonk, NY, USA). Data has been presented as means and standard deviations. The Shapiro–Wilk test was used to confirm normality, and the Levene test was used to verify the homogeneity of variances. A two-way repeated measures ANOVA (2 groups × 2 times) was used to compare results on the passing, speed shooting, dribbling, and defensive movement tests. Conquering the ball, receiving the ball, losing the ball, successful shorts, the volume of play, and efficiency indexes were compared using a multivariate analysis of variance (MANOVA). For the variables (Offensive and the number of attack balls), an analysis of covariance (ANCOVA) was performed with a pre-test as the covariate. When a significant difference was reported, a Bonferroni post hoc test was used to detect differences in means. Partial eta squared (η_p_^2^) effect size values were reported and classified as 0.01 = small, 0.06 = medium, 0.14 = large [51]. Moreover, standardized effect size analysis (Cohen’s *d*) was used to interpret the magnitude of differences between variables and considered as: trivial (≤0.20); small (0.20 < *d* ≤ 0.60); moderate (0.60 < *d* ≤ 1.20); large (1.20 < *d* ≤ 2.0); very large (2.0 < *d* ≤ 4.0); and extremely large (>4.0) [52]. In addition, the upper and lower 95% confidence intervals of the difference (95%CI_dif_) were calculated for the corresponding variation. The level of statistical significance was set at *p* ≤ 0.05.

## 3. Results

Table 2 reported results for normality from Shapiro–Wilk test and homogeneity of variance assessed by Levene’s test for dependent variables assessed by the basketball skill test (AAHPERD).

Table 3 reported results for normality from Shapiro–Wilk test and homogeneity of variance assessed by Levene’s test for dependent variables assessed by the three vs. three small-sided games.

Table 4 presents the performances recorded in both the experimental and control groups before and after the intervention period for the Basketball Skill Test.

For the passing test, there was a main effect of time (*F*_1,18_ = 55.27; *p* < 0.001; η_p_^2^ = 0.754) with values higher after the intervention period compared to before the intervention (95%CI_dif_ = 8.9 to 15.8; *d* = 1.27; *p* < 0.001). There was a main effect of group (*F*_1,18_ = 6.38; *p* = 0.021; η_p_^2^ = 0.262) with values higher for VMG compared to CG (95%CI_dif_ = 1.5 to 16.7; *d* = 0.87; *p* = 0.021). For the speed shooting test, there was a main effect of time (*F*_1,18_ = 20.25; *p* < 0.001; η_p_^2^ = 0.529) with values higher after the intervention compared to before intervention (95%CI_dif_ = 3.9 to 10.9; *d* = 1.03; *p* < 0.001). For the dribbling test, there was a main effect of time (*F*_1,18_ = 16.10; *p* = 0.001; η_p_^2^ = 0.472) with lower values recorded after the intervention period compared to before the intervention period (95%CI_dif_ = −4.0 to −1.3; *d* = −1.04; *p* = 0.001). For the defensive movement test, there was a main effect of time (*F*_1,18_ = 117.9; *p* < 0.001; η_p_^2^ = 0.868) with lower values recorded after the intervention period compared to before (95%CI_dif_ = −5.5 to −3.7.7; *d* = −2.36; *p* < 0.001).

Table 5 presents the performances recorded on both the experimental and control groups before and after the intervention period during the small, sided game. For offensive balls post-intervention, there was a main effect of group (*F*_1,17_ = 11.73; *p* = 0.003; η_p_^2^ = 0.408), with performance values being higher for VMG compared to CG (95%CI_dif_ = 1.6 to 6.8; *d* = 1.81; *p* = 0.003). For the number of attack balls index post-intervention, there was a main effect of group (*F*_1,17_ = 16.66; *p* = 0.001; η_p_^2^ = 0.495), with performance values recording higher for VMG compared to CG (95%CI_dif_ = 3.1 to 9.8; *d* = 0.28; *p* = 0.001).

## 4. Discussion

The aim of the present study was to investigate the effects of adding video modeling during four weeks of a basketball training program on individual technical performances assessed by the Basketball Skill Test-American Alliance for Health, Physical Education, Recreation and Dance (i.e., passing, shooting, dribbling, and defense) and collective aspects assessed by the three vs. three small-sided games (i.e., conquering the ball, receiving the ball, losing the ball, successful shorts, the volume of play, the number of attacking balls, volume of play, and the efficiency index) among young novice basketball players.

The results revealed that the VMG reported a significant improvement over the control group on the passing test. Nevertheless, the dribbling and the defensive movement test showed lower values after the intervention, indicating a potentially negative effect on these skills. Additionally, regarding collective game performance, results showed that VMG resulted in higher offensive balls, number of attack balls index, and lower ball loss compared to the control group and that the efficiency index was higher for VMG after the intervention compared to before. These results partially confirm our hypothesis.

The results recorded in the present study confirm those reported in several previous studies that confirmed that incorporating VM was a good strategy to induce beneficial improvements in terms of specific skills in both individuals (e.g., gymnastics, weightlifting) [15,16,53], and team sports such as soccer and basketball [54,55,56]. Specifically, in basketball, passing skills were found to be higher in VMG compared to CG. While these results cannot absolutely confirm the effectiveness of VMG compared to CG in improving this technique (i.e., no interaction effect between the groups and the time of measurement), it can open a window to focus more on this strategy as a way to improve basketball skills within young players. In this consideration, results from previous studies supported the efficacy of using video modeling in improving passing skills in school settings [20,22]. The improvement in the VMG can be explained by the state of motivation within these young players when watching skilled athletes executing this technique in addition to the importance of this technique which is one of the most important technical aspects of basketball performance [57,58,59]. This perspective conforms to social learning theory, which suggests that individuals may learn by observing others’ behaviors and the consequences of that behavior [3,60]. However, it should be noted that the dribbling and defensive movement tests showed lower values after the intervention, indicating a potentially negative effect on these skills; this finding is not consistent with previous research which showed that video modeling was effective in improving basketball players’ performance in dribbling and defensive skills (e.g., [61]). One possible explanation for this finding is that the video modeling intervention may have overemphasized passing skills at the expense of other important technical skills. It is also possible that the relatively short duration of the intervention (4 weeks) was not sufficient to produce significant improvements in all technical skills.

More interestingly, in terms of collective performance, the results showed that VMG increased the number of offensive balls and the index of the number of attacking balls, and reduced ball losses compared to the control group. In addition, the efficiency index was higher for VMG after the intervention than before. These results suggest that video modeling can be an effective tool for improving collective game performance in young novice basketball players. This is consistent with previous research that has shown the benefits of video modeling to improve team cohesion, communication, and decision-making [62,63]. Contrary to our results, Panchuk et al. [19] showed that following an immersive video intervention, individual technical performances (i.e., number of successful passes, assists, hockey assists, contested shots, deflected passes, passing turnovers, and dribbling turnovers) recorded during small-sided games did not improve compared to the control group [19]. This inconsistent result may be attributed to the difference in the competition level of participants (elite vs. novice players in the current study), type of the video modeling used, and the duration of the training intervention. Nevertheless, the results of the present study underline how much is important to assist young novice basketball players during their technical-tactical training processes using technology such as video modeling. The decrease in lost balls during small-sided games may be attributed to passing skill improvement which was assessed using AAHPERD [43].

### Strengths and Limitations

The main strengths of the study were that we used objective measures of performance (e.g., passing test, offensive balls, loss of the ball) rather than relying on subjective assessments. In addition, the study used a training program designed in an ecologically valid environment with real-field-based learning experiences that could easily be replicated by coaches and trainers. However, we acknowledge some limitations of this study. First, the participants were novice players, which may restrict the generalization of the results. Again, the study sample was mainly males, and this calls for further comparative investigations across gender to ascertain motor skill learning variations. Moreover, the duration of the study was only four weeks, which may not have been long enough to induce significant improvements in other individual techniques that may require more training periods to be improved. Further, a retention test was also not included as part of the experimental protocol which restricts the long-term motor learning effects of an intervention. Usually, a standardized motor learning process has three distinct phases: acquisition, retention, and a transfer phase, where teachers are encouraged to intensify the complexity of preceding motor tasks and/ or situations (i.e., simple to complex) [64,65]. Future studies could incorporate these measures using longitudinal designs to investigate patterns across gender.

## 5. Conclusions

The results of the present study showed that a training program based on video modeling improved performance on the passing test, as well as offensive balls. Additionally, the number of post-intervention offensive ball indices was higher for VMG than for CG. For ball loss, VMG induced lower values than CG post-intervention. The efficiency index was higher after training than before training for VMG. The results of the study may be a useful tool for coaches in designing effective training programs that seeks to develop the individual and collective technical skills of their players which are keys to success in basketball. As well, four weeks of video modeling with a frequency of four sessions per week was effective to improve some technical skills, while others were not improved. Further studies are required to evaluate the progression of young players following a training program using video modeling for an extended period.

## Figures and Tables

**Table 1:** Anthropometric measurements of the CG and VMG groups (*n* = 10 each): Age, body mass, and height.

	CG (*n* = 10)	VMG (*n* = 10)
Age (year)	12 ± 0.7	12.5 ± 0.5
Body mass (kg)	47 ± 11	51 ± 16
Height (cm)	153 ± 10.5	158 ± 9.5

**Table 2:** Normality and homogeneity of variance values for dependent variables assessed by the basketball skill test.

		Shapiro–Wilk Test		Levene’s Test	
		W	p	F	p
Passing	Pre	0.956	0.460	1.468	0.351
	Post	0.950	0.367	0.003	0.959
Shooting	Pre	0.958	0.510	0.001	0.985
	Post	0.979	0.926	1.292	0.271
Defense	Pre	0.949	0.348	0.116	0.737
	Post	0.956	0.464	0.103	0.752
Dribbling	Pre	0.966	0.674	0.042	0.840
	Post	0.964	0.630	0.060	0.809

W: Shapiro–Wilk statistic, F: Levene’s test statistic.

**Table 3:** Normality and homogeneity of variance values for dependent variables assessed by the 3 vs. 3 small-sided games.

	Shapiro–Wilk Test		Levene’s Test	
	W	p	F	p
CB	0.965	0.251	0.913	0.444
RB	0.973	0.451	1.170	0.334
LB	0.942	0.338	3.082	0.032
SS	0.964	0.640	0.403	0.752
PB	0.949	0.072	0.593	0.624
OB	0.983	0.814	0.006	0.940
AB	0.971	0.378	0.993	0.332
Efficiency	0.969	0.341	0.393	0.759

W: Shapiro–Wilk statistic, F: Levene’s test statistic; CB: conquering the ball; RB: receiving the ball; LB: loosed ball; OB: offensive ball; SS: successful shot; AB: attacking ball; PB: volume of play.

**Table 4:** Technical performances recorded in the basketball skill test for both video modeling (VMG) and control groups (CG) before and after the intervention period (Values are mean ± SD; *n* = 20).

	CG (*n* = 10)		VMG (*n* = 10)	
	Pre-Test	Post-Test	Pre-Test	Post-Test
Passing (point)	42.20 ± 12.73	51.80 ± 6.46 *	48.60 ± 8.06+	63.70 ± 6.63 *+
Shooting (point)	22.9 ± 7.32	31.70 ± 6.46 *	21.64 ± 6.64	27.70 ± 8.68 *
Defense (s)	27.40 ± 2.40	23.47 ± 1.60	28.82 ± 2.05	23.51 ± 1.74
Dribbling (s)	26.35 ± 3.66	23.74 ± 1.84 *	27.13 ± 3.07	24.43 ± 1.35 *

* Indicates significant difference from pre-test (*p* < 0.05); + indicates significant difference from CG (*p* < 0.05).

**Table 5:** Technical performances recorded in the small, sided games for both the video modeling group (VMG) and control group (CG) before and after the intervention period (Values are mean ± SD; *n* = 20).

	CG (*n* = 10)		VMG (*n* = 10)	
	Pre-Test	Post-Test	Pre-Test	Post-Test
CB	7 ± 3	7 ± 3	6 ± 3	6 ± 2
RB	10 ± 2	8 ± 2	8 ± 3	9 ± 2
LB	6 ± 2	8 ± 2 †	6 ± 3 *	4 ± 1 *+
OB	14 ± 5	12 ± 3	7 ± 3	12 ± 4 *
SS	1 ± 2	1 ± 2	1 ± 1	3 ± 2
AB	15 ± 6	14 ± 4	8 ± 4	15 ± 6 *
PB	16 ± 4	15 ± 4	14 ± 5	16 ± 2
Efficiency Index	1.3 ± 0.6	1.1 ± 0.4	0.97 ± 0.4	1.5 ± 0.5 ¶

* Indicates a significant difference from CG (*p* < 0.05); + indicates lower values after the intervention compared to CG (*p* < 0.05); † indicates higher values for CG post-intervention compared to before (*p* < 0.05); ¶ indicates higher values for VMG post-intervention compared to pre (*p* < 0.05). CB: conquering the ball; RB: receiving the ball; LB: loosed ball; OB: offensive ball; SS: successful shot; AB: attacking ball; PB: volume of play.

## Data Availability

The original contributions presented in the study are included in the article, further inquiries can be directed to the corresponding author.
